# Aortic Arch Calcification on routine Chest Radiography is Strongly and Independently Associated with Non-Dipper Blood Pressure Pattern

**DOI:** 10.36660/abc.20190790

**Published:** 2020-01

**Authors:** Rui Póvoa

**Affiliations:** Universidade Federal de São Paulo, São Paulo, SP - Brazil

**Keywords:** Thoracic, Aorta/physiopathology, Calcification, Calcinosis, Cardiomyopathies, Hypertension, Ventricular Function, Left, Antihypertensive Agents, Blood Pressure Monitoring Ambulatory, Heart Rate

Arterial hypertension is the most prevalent chronic disease worldwide, and it is estimated that in Brazil, according to the Vigitel study of 2018, the Brazilian population has 24.7% of hypertensive individuals.^1^ It is the most important risk factor for all cardiovascular diseases, being one of the main factors responsible for stroke, coronary artery disease, heart failure, renal failure, and so on.

Blood pressure is not simply a biological phenomenon characterized by two numbers expressed in mmHg that reflect systolic and diastolic pressure. It is a multiform event resulting from the hydrodynamic action of a complex fluid that exerts force on a compliant vascular wall, with elastic properties governed by multiple forces arising from vascular structures (muscle cells, extracellular matrix, elastic fibers, etc.). This vector wave of blood follows the laws of sympathetic and parasympathetic interaction, varying more or less depending on the conditions of each patient. Therefore, the simple measurement of blood pressure in the office does not reflect the reality of the patient's true daily life.

The several measurements obtained during the 24-hour period have a better meaning in the global context evaluation and can discriminate the blood pressure behavior in the different phases of the day and, especially, during sleep. Ambulatory blood pressure monitoring (ABPM) has a better correlation with all cardiovascular events compared to simple office measurements. The pressure varies widely during the 24-hour period, and during sleep, in individuals considered normal, there is a reduction between 10 and 20% of daytime values. In some studies, the absence of this physiological dipping during sleep, which we call “non-dipper”, shows a positive association with an increase in target-organ damage and mortality.^1,2^

This concept that hypertensive individuals (as well as normotensive ones) who do not have a physiological pressure dipping during sleep (non-dipper), are subject to a worse prognosis has been a consensus for some time, well evidenced in several studies; however, the intrinsic mechanisms that lead to a higher risk are not yet fully known.^3-5^ In clinical practice, hypertensive patients with the non-dipper pattern usually have some other comorbidity, leading to the suspicion of secondary causes for blood pressure elevation and/or obstructive sleep apnea.

The usefulness of ABPM is quite broad. The European guideline for hypertension very objectively suggests performing out-of-office blood pressure measurements, supplemented with in-office measurements for diagnosing hypertension.^6^

The phenomenon of white coat hypertension and the white coat effect are common and may lead to case wrong conduct and management. In the first, there is an erroneous diagnosis of hypertension and in the presence of the white coat effect, one can start the medication incorrectly, with harm to the patient. These are situations where ABPM is critical for the diagnosis.

Another important and not infrequent aspect is masked hypertension, where the patient has normal office pressure values and levels outside the office are elevated. This situation can be correctly assessed only by out-of-office measurements and represents a huge danger, as target-organ damage is more intense and occurs earlier.^7^

The assessment of out-of-office BP can also be attained by the much more inexpensive and affordable home blood pressure measurement (HBPM), which shows a good correlation with ABPM measurements. However, it does not evaluate BP during sleep, the fundamental period of the biological cycle. That is why ABPM remains the gold standard for out-of-office measurements.^8^

The assessment of sleep, of the phenomena that occur before waking up and after awakening are also very important for a more precise stratification of the risk of cardiovascular events. Kazuomi Kario was one of the pioneers in assessing the effect of arousal on cardiovascular risk, finding greater target-organ damage in those with more intense and sustained blood pressure response.^9^

Nakanishi et al.^10^ found that patients who experienced BP increases during sleep rather than the physiological reduction were more subject to cardiovascular events. Studying a population of 828 patients through brain imaging tests, such as MRI, they found that elevated systolic pressure during sleep was associated with subclinical brain disease.^10^

In the study by Adar A. et al.,^11^ they found that patients who had aortic arch calcification on routine chest X-ray showed an association with the non-dipper pattern in ABPM.^11^ The authors started by detecting a target-organ lesion, aortic atherosclerosis, in a routine exam to look for changes in blood pressure physiology and eventually diagnose or correct other problems.

This association is important, because we can verify the entire aortic arch calcification in the search for non-dippers and, thus, better typify the behavior of arterial hypertension.

Chest X-ray, used in many clinical situations, is an inexpensive, practical exam and, when performed for some other reason, it can also be used to screen for non-dippers, as demonstrated in this study. This may be beneficial to the patient, since secondary causes of hypertension, including obstructive sleep apnea, are frequent.

The pathophysiological justification of this association, between the aortic artery calcification and the non-dipping of pressure during sleep, remains unknown. The authors mention the association of this calcification with pineal gland calcification and melatonin reduction, which plays an important role in sleep regulation.^12^ Moreover, it also participates in the autonomic regulation with greater accentuation of the parasympathetic system with direct vasodilating effects, thereby reducing blood pressure.^13^

The finding of calcification in the aortic arch allowing the diagnosis of the presence of atherosclerosis immediately changes the cardiovascular risk, wherein clinical care should be more intense and thus improve our clinical practice.

Although there are not robust clinical trials evaluating chromotherapy and the influence of antihypertensive drugs on the non-dipper pattern yet, it is sensible and intuitive that this should be done, when there is no objective cause for no reduction of blood pressure during sleep. In hypertensive patients, the change in the time of medication may change the pattern of nocturnal dipping and possibly benefit the patient.^14,15^

An adequate BP control in the early hours of the morning means the use of drugs with adequate 24-hour coverage; however, some medications do not provide this effect. Changing the time when the patient takes the drug, particularly to the afternoon, may result in a pressure behavior closer to the normal circadian rhythm.

We still have a long road ahead to assess blood pressure behavior, but some light is already emerging to unravel the mechanisms implicated in this complex web of hypertensive disease etiology.
